# Supplementary material for: The environmental sustainability of digital content consumption
Source: Nat Commun. 2024 May 2;15:3724. doi: 10.1038/s41467-024-47621-w (PMC11066053; doi:10.1038/s41467-024-47621-w)
Supplement: Supplementary file 1 — Supplementary Information [file 41467_2024_47621_MOESM1_ESM.pdf]

The environmental sustainability of digital content consumption

Supplementary Information

Robert Istrate[1,2], Victor Tulus[1], Robert N. Grass[1], Laurent Vanbever[3], Wendelin J. Stark[1], Gonzalo Guillén-Gosálbez[1*]

[1] Institute for Chemical and Bioengineering, Department of Chemistry and Applied Biosciences, ETH Zürich, Vladimir-Prelog-Weg 1, Zürich 8093, Switzerland.
[2] Institute of Environmental Sciences (CML), Leiden University, Einsteinweg 2, 2333 CC Leiden, the Netherlands.
[3] Computer Engineering and Networks Laboratory, Department of Information Technology and Electrical Engineering, ETH Zürich, Gloriastrasse 35, Zürich 8092, Switzerland.
*Corresponding author: gonzalo.guillen.gosalbez@chem.ethz.ch

This document provides additional results, describes data sources, and discusses additional methodological assumptions and limitations.

Table of Contents

1 Supplementary results 3
1.1 Climate impacts under different carbon budgets 4
1.2 Additional breakdown of impacts 6
1.3 Environmental impacts considering global data centres powered by renewable electricity 8
1.4 Environmental impacts considering changes in digital content consumption patterns 10
1.5 Uncertainty analysis 13
2 Life cycle inventory data 15
3 Earth's carrying capacity 24
4 Methodological assumptions, limitations, and future work 29
5 Supplementary references 32

1 Supplementary results

This section provides additional results omitted in the main manuscript. These include: i) climate impacts of digital content consumption under different carbon budgets (**Supplementary Fig. 1**); ii) breakdown of impacts embodied in end-user devices (**Supplementary Fig. 2**) and breakdown of total impacts by digital content (**Supplementary Fig. 3**); iii) environmental impacts considering global data centres powered by renewable electricity (**Supplementary Fig. 4** and **Supplementary Fig. 5**); iv) environmental impacts considering changes in digital content consumption patterns (**Supplementary Fig. 6** and **Supplementary Fig. 7**); v) uncertainty analysis results based on Monte Carlo simulation (**Supplementary Fig. 8**).

1.1 Climate impacts under different carbon budgets

The results presented in the main manuscript consider a stringent carbon budget (501 kg CO_2 per capita per year) consistent with a high likelihood (67%) of limiting global warming to 1.5 °C above the pre-industrial level. We generated additional results for different temperature limit targets (i.e., 1.5 °C, 1.7 °C, and 2.0 °C) and likelihoods of reaching these targets (i.e., 17%, 33%, 50%, 67%, and 83%) based on the Intergovernmental Panel on Climate Change (IPCC) Sixth Assessment Report (AR6)[1]. The cumulative carbon budgets over the period 2020-2100 range from 300 Gt CO_2 for an 83% chance of limiting global warming to 1.5 °C to 2,300 Gt CO_2 for a 17% chance of limiting global warming to 2 °C (AR6 Table SPM.2, p. 41 "Estimates of historical emissions and remaining carbon budgets"[1]). Supplementary Table 1 shows the estimated per capita carbon budgets following the equal share per capita approach as described in the Methods section of the main manuscript. Furthermore, Supplementary Fig. 1 depicts the corresponding share occupied by digital content consumption, which varies from 7% to 55%.

Supplementary Table 1. Estimated per capita carbon budgets from the beginning of 2020 based on IPCC AR6[1]. The values represent the remaining kg of CO_2 per capita per year as of 2020 until global net zero CO_2 emissions are reached.

Temperature limit	Likelihood of limiting global warming to temperature limit				
	17%	33%	50%	67%	83%
**1.5 °C**	1,126	813	626	501	375
**1.7 °C**	1,815	1,314	1,064	876	688
**2.0 °C**	2,878	2,127	1,689	1,439	1,126

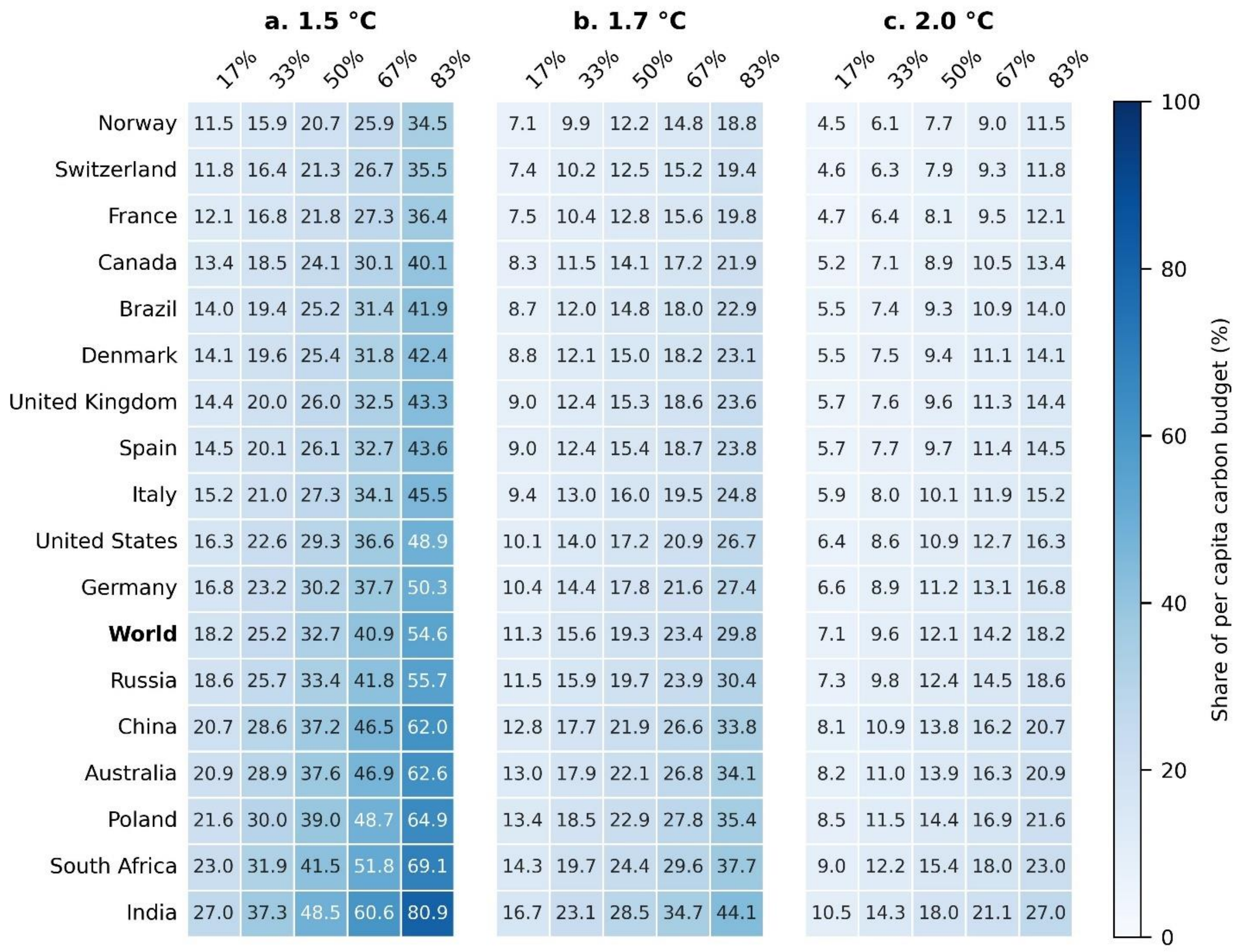

Supplementary Fig. 1. Share of per capita carbon budget required by digital content consumption considering carbon budgets consistent with different temperature limit targets (1.5 °C, 1.7 °C, and 2.0 °C) and likelihoods of achieving these targets (17%, 33%, 50%, 67%, and 83%). Impacts for a user archetype representing the global average consumption patterns across all Internet users and using the global average electricity mix. Rows in the heatmap correspond to different user locations ranked according to the required share (the global average is labelled in bold).

1.2 Additional breakdown of impacts

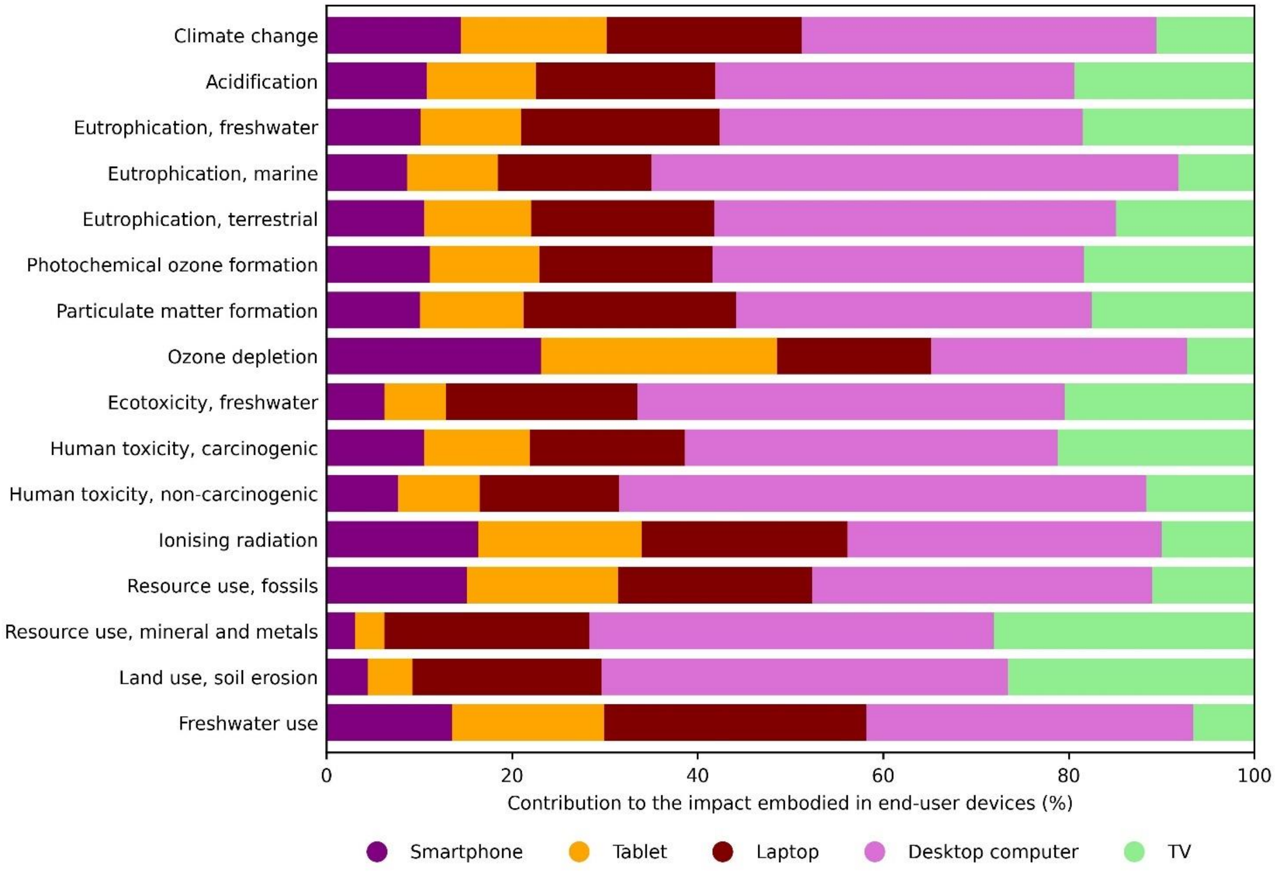

Supplementary Fig. 2. Life cycle environmental impacts embodied in end-user devices breakdown by device type. Impacts for a user archetype representing the global average consumption patterns across all Internet users and using the global average electricity mix. Embodied refers to impacts from raw materials extraction, manufacturing, distribution, and end-of-life management. The desktop computer is the largest contributor (between 28% and 58% of embodied impacts, depending on the category), whereas the smartphone is the lowest one even though it is the most used device (53% of the time spent on digital content).

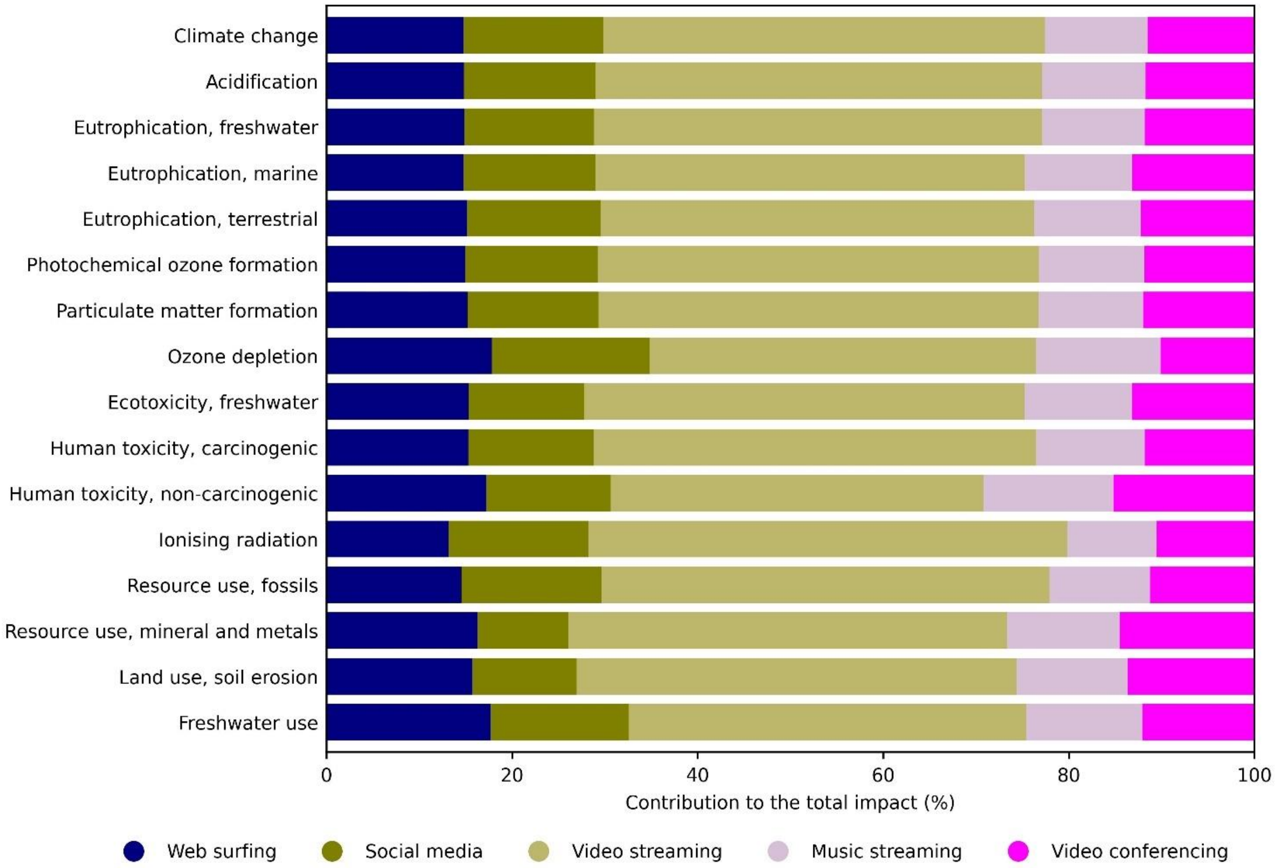

Supplementary Fig. 3. Life cycle environmental impacts of digital content consumption breakdown by digital content. Impacts for a user archetype representing the global average consumption patterns across all Internet users and using the global average electricity mix. Video streaming generates on average between 42% and 51% of the total impacts, while web surfing, social media, music streaming, and video conferencing contribute each with 10−18%.

1.3 Environmental impacts considering global data centres powered by renewable electricity

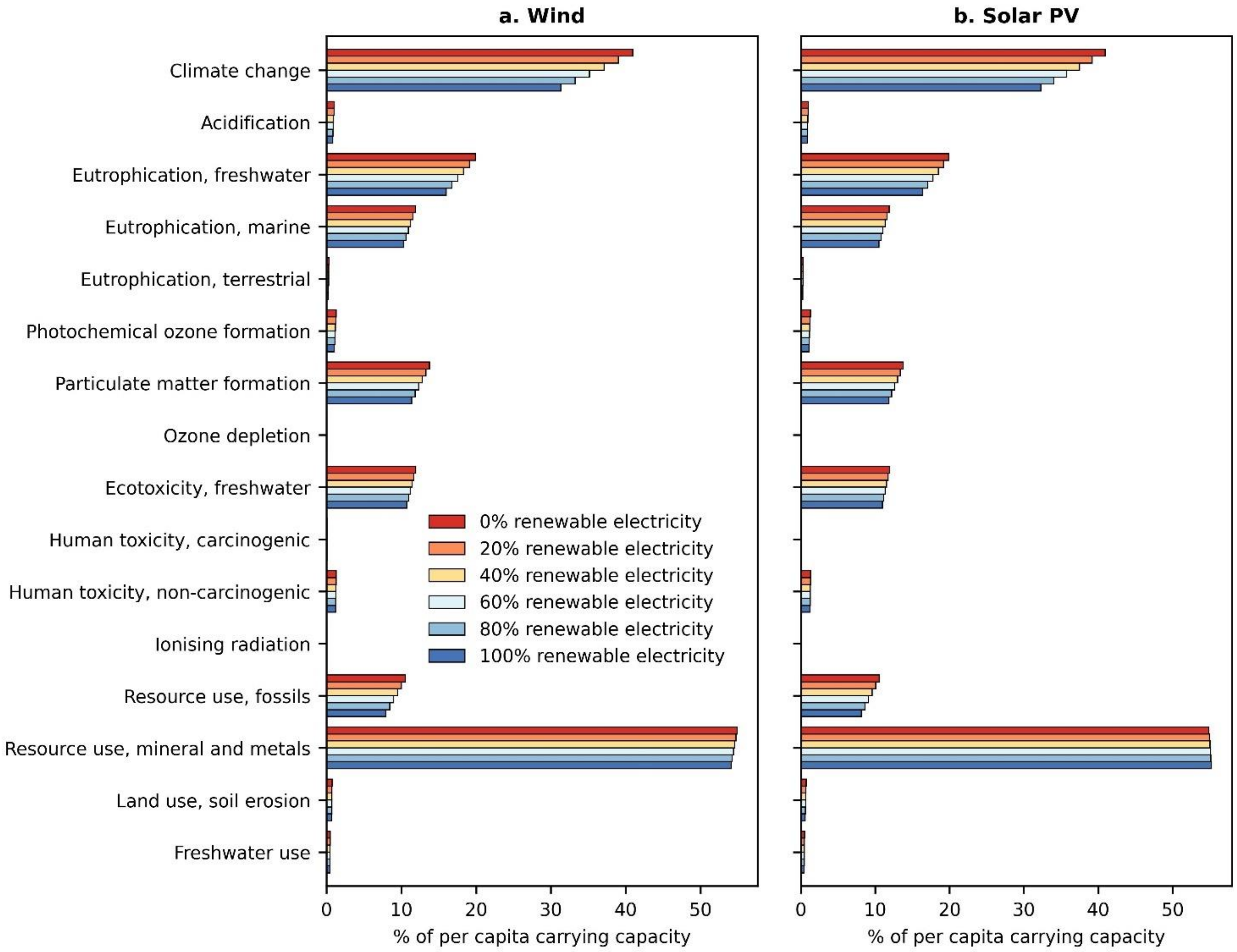

Supplementary Fig. 4. Share of per capita carrying capacity required by digital content consumption over a year considering that global data centres are partially powered by (a) wind and (b) solar PV. The "100% renewable electricity" scenario corresponds to a hypothetical scenario in which global data centres are entirely powered by renewable electricity, while the "0% renewable electricity" scenario corresponds to the default assumption in which global data centres are entirely powered by the regional electricity mixes. Impacts for a user archetype representing the global average consumption patterns across all Internet users and using the global average electricity mix (for charging end-user devices).

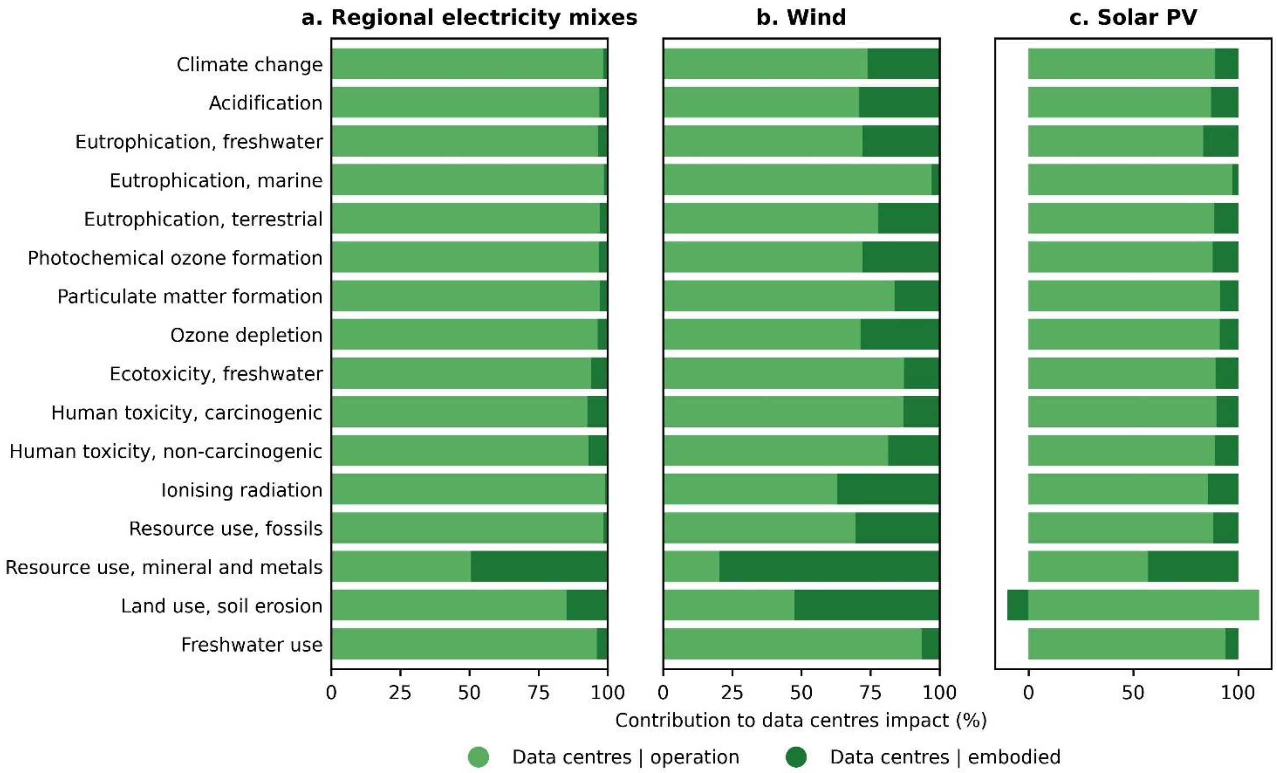

Supplementary Fig. 5. Breakdown of data centres impacts considering that they are powered by (a) the regional electricity mixes, (b) wind, and (c) solar PV. Impacts for a user archetype representing the global average consumption patterns across all Internet users and using the global average electricity mix (for charging end-user devices).

1.4 Environmental impacts considering changes in digital content consumption patterns

In the main manuscript we present the environmental impacts of digital content consumption considering a user archetype representing the global average consumption patterns across all Internet users. Here, we present the results for two alternative user archetypes that differ in their preferences. To provide a lower bound, Supplementary Fig. 6 shows the impacts for a user who only uses a smartphone –the end-user device with the lowest electricity intensity–, watches video streaming at low quality (480p resolution), listens music at low quality, and joins online meetings with audio only. Moreover, to provide an upper bound, Supplementary Fig. 7 shows the impacts for a user who watches video streaming at ultra-high definition (4K resolution), listens music at high quality, and joins online meetings with high quality video.

	Carbon footprint	Climate change	Acidification	Eutrophication, freshwater	Eutrophication, marine	Eutrophication, terrestrial	Photochemical ozone formation	Particulate matter formation	Ozone depletion	Ecotoxicity, freshwater	Human toxicity, carcinogenic	Human toxicity, non-carcinogenic	Ionising radiation	Resource use, fossils	Resource use, mineral and metals	Land use, soil erosion	Freshwater use
Norway	68	11.9	0.3	6.3	3.5	0.1	0.4	4.2	0.0	3.9	0.0	0.4	0.0	3.2	17.9	0.2	0.2
Switzerland	70	12.3	0.3	6.4	3.5	0.1	0.4	4.2	0.0	3.9	0.0	0.4	0.0	3.8	18.0	0.2	0.2
France	71	12.6	0.3	6.4	3.7	0.1	0.4	4.5	0.0	3.9	0.0	0.4	0.1	5.5	18.0	0.2	0.3
Canada	78	14.0	0.3	7.9	3.8	0.1	0.4	4.3	0.0	4.0	0.0	0.4	0.0	4.1	17.8	0.2	0.3
Denmark	84	14.9	0.3	7.8	3.9	0.1	0.4	4.6	0.0	4.1	0.0	0.4	0.0	3.9	18.1	0.2	0.2
Brazil	84	14.7	0.4	6.4	3.9	0.1	0.4	5.8	0.0	4.0	0.0	0.4	0.0	3.7	17.9	0.7	0.2
United Kingdom	85	15.2	0.3	6.6	3.9	0.1	0.4	4.5	0.0	4.0	0.0	0.4	0.0	4.8	18.0	0.2	0.2
Spain	86	15.3	0.4	7.1	4.3	0.1	0.5	4.7	0.0	4.1	0.0	0.4	0.0	4.6	18.0	0.2	0.2
Italy	90	16.0	0.4	6.9	4.0	0.1	0.5	4.6	0.0	4.1	0.0	0.4	0.0	4.3	18.0	0.2	0.2
United States	96	17.2	0.4	9.2	4.0	0.1	0.4	4.8	0.0	4.9	0.0	0.4	0.0	4.8	18.0	0.2	0.3
Germany	99	17.8	0.4	12.2	4.2	0.1	0.4	4.5	0.0	4.4	0.0	0.4	0.0	4.6	18.2	0.2	0.3
**World**	109	19.3	0.5	8.9	4.8	0.1	0.6	6.3	0.0	4.6	0.0	0.4	0.0	5.0	18.0	0.3	0.3
Russia	112	19.8	0.5	9.5	4.6	0.1	0.5	4.8	0.0	4.3	0.0	0.4	0.0	5.8	18.0	0.2	0.3
Australia	122	22.3	0.5	18.4	5.5	0.1	0.6	4.6	0.0	4.8	0.0	0.5	0.0	5.1	18.0	0.3	0.3
Poland	127	23.2	0.6	16.4	5.5	0.2	0.7	5.0	0.0	4.9	0.0	0.6	0.0	5.4	18.0	0.3	0.3
China	128	22.1	0.6	7.7	5.8	0.2	0.7	10.4	0.0	4.8	0.0	0.5	0.0	5.0	18.0	0.2	0.3
South Africa	133	24.7	1.0	12.7	7.6	0.3	1.0	5.5	0.0	4.1	0.1	0.6	0.0	6.2	18.0	0.4	0.2
India	157	29.1	0.6	12.6	6.4	0.2	0.8	5.7	0.0	4.8	0.0	0.6	0.0	6.9	18.0	0.4	0.3

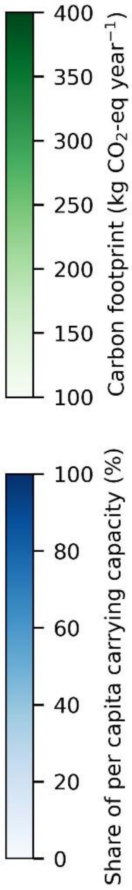

Supplementary Fig. 6. Carbon footprint and share of per capita carrying capacity occupied by digital content consumption for a hypothetical user who only uses a smartphone –the end-user device with the lowest electricity intensity–, watches video streaming and listens music at low quality, and joins online meetings with audio only. Other assumptions are the same as for the global average user. Rows in the heatmap correspond to the electricity mix of various countries ranked according to the carbon footprint (the global average electricity mix is labelled in bold).

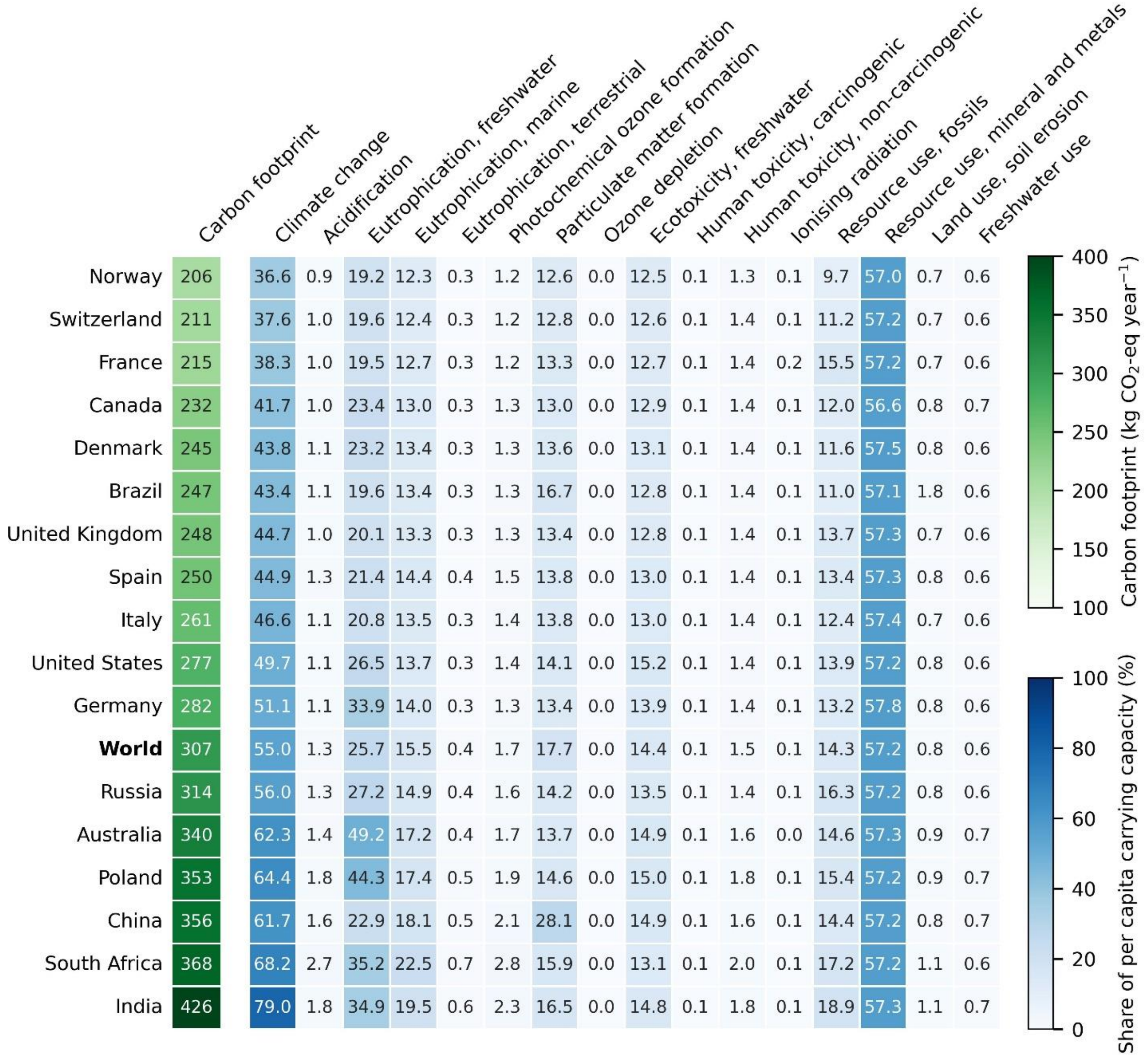

Supplementary Fig. 7. Carbon footprint and share of per capita carrying capacity occupied by digital content consumption for a hypothetical user who watches video streaming at ultra-high definition (i.e., 4K resolution), listens music at high quality, and joins online meetings with high quality video. Other assumptions are the same as for the global average user. Rows in the heatmap correspond to the electricity mix of various countries ranked according to the carbon footprint (the global average electricity mix is labelled in bold)

1.5 Uncertainty analysis

The uncertainties linked to the LCA results due to uncertainties in the life cycle inventory (LCI) data was assessed by error propagation via the Monte Carlo sampling method based on 1,000 runs. For each run, LCI data values were randomly sampled according to their probability distribution and the life cycle environmental impacts of digital content consumption for the global average user were quantified. The impact value for each run and category was then compared against the corresponding per capita Earth's carrying capacity. Probability distributions were determined based on the literature for Internet network components data (Supplementary Tables 5-8) and the ecoinvent database v3.8[3] for background inventory data (i.e., manufacturing processes, power generation, etc.). It should be noted that the ecoinvent database uses the Pedigree matrix to model the uncertainties affecting the inventory data based on five independent data features: "reliability", "completeness", "temporal correlation", "geographic correlation", and "further technological correlation".

The results produced by the Monte Carlo simulation for the global average user are reported in Supplementary Fig. 8. The results show that the required share of the per capita carrying capacity for acidification, terrestrial eutrophication, photochemical ozone formation, ozone depletion, carcinogenic human toxicity, ionising radiation, and land and freshwater use remains negligible, even after considering the uncertainties in the LCI data (probabilities below 5% of shares above 2%). Regarding the most critical impact categories, the largest dispersion is observed for mineral and metals resources depletion, freshwater eutrophication, particulate matter formation, non-carcinogenic human toxicity, and climate change. The deterministic impact values presented in the main manuscript are close to the lower bound of the 95% uncertainty rage (defined as the 2.5-97.5th percentiles calculated with pandas v1.5.3), mainly owing to the shape of the lognormal distributions used to model background data.

Moreover, these results suggest that the required share of the carrying capacities may be even higher. Based on this analysis, we can conclude that the potential impact of uncertainties in LCI data on the outcome of our study might be very limited, i.e., that digital content consumption would still require a substantial share of the per capita Earth's carrying capacity across several impact categories considering current infrastructure.

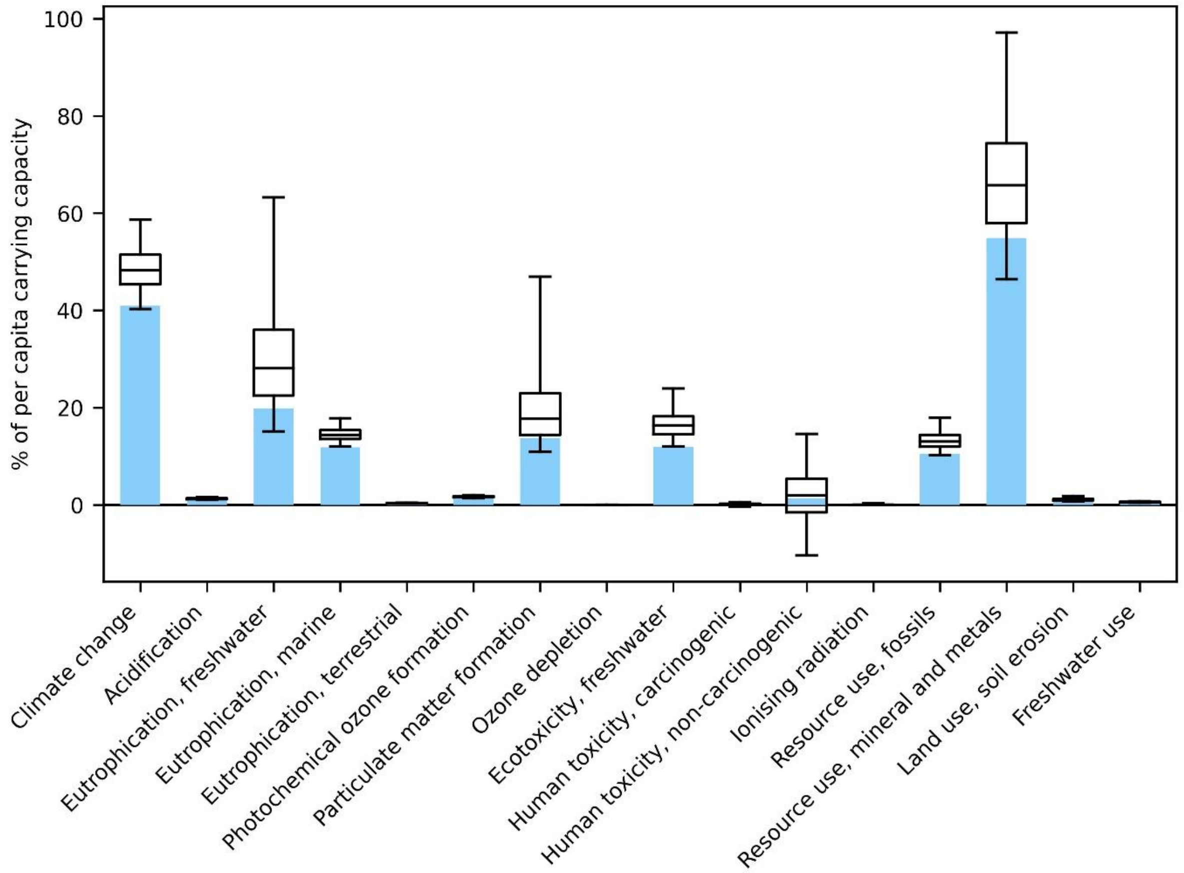

Supplementary Fig. 8. Monte Carlo simulation results showing the per capita carrying capacity required by digital content consumption over a year (n=1,000 runs). Impacts for a user archetype representing the global average consumption patterns across all Internet users. The bottom and top edges of the box indicate the 25th and 75th percentiles, respectively. The line within the box indicates the median value. The whiskers show the 2.5^{th} and 97.5^{th} percentiles, corresponding to the 95% uncertainty range. The bars represent the deterministic impacts as presented in the main manuscript. Note that the deterministic values depart from the median values since parameters were generally modelled with skewed distributions. According to ref.[4], it is a known issue in LCA that the mean and median values obtained from Monte Carlo simulations are consistently higher than the deterministic impacts.

2 Life cycle inventory data

This section presents the life cycle inventory (LCI) data used to quantify the environmental impacts of digital content consumption. **Supplementary Table 2** and **Supplementary Table 3** show the consumption patterns of the user archetype concerning the annual digital content consumption, quality/resolution, and end-user devices used. **Supplementary Table 4** reports the electricity intensities used to model electricity consumption during the operation of the Internet infrastructure. **Supplementary Table 5** and **Supplementary Table 6** shows the infrastructure-related inventory data. Finally, **Supplementary Table 7** includes the data traffic demand of digital content. The LCI data is provided in a format that can be directly imported into Brightway2 at ref.[5].

Supplementary Table 2. Annual digital content consumption patterns that define the user archetype.

Digital content	Daily consumption	Annual consumption	Assumptions and data source
Web surfing	2h 00min	730h	Assumed equal to the daily time spent reading online media by Internet users aged 16 to 64 in 2022[6].
Social media	2h 27min	894h	Average daily time spent with social media by Internet users aged 16 to 64 in 2022[6].
Video streaming	2h 17min	833h	According to recent statistics, an average person spends 16 hours per week on video streaming[*].
Music streaming	1h 33min	566h	Average daily time spent with music streaming by Internet users aged 16 to 64 in 2022[6].
Video conferencing	0h 34min	207h	A recent survey revealed that about 46% of the respondents spend less than 4 hour per week on video conferencing, while 37% spend between 4 and 12 hours per week[†]. We assume 4 hours per week as representative for an average person

[*] Duoplus: Online Video Consumption Is Booming (2021)
[†] Dialpad: The state of video conferencing 2022

Supplementary Table 3. Digital content access by end-user device. The percentages represent the share of the annual consumption that the digital content is accessed with each device type. For example, social media is accessed through a smartphone 83% of the time, while tablet, laptop, and desktop computer each account for 5.67% of the time.

Digital content	Quality/resolution	Smartphone	Tablet	Laptop	Desktop computer	TV 720p	TV 1080p	Assumptions and data source
Web surfing	–	50%	15%	25%	10%	–	–	Based on a survey of the device preferences for Internet browsing or surfing among Internet users in the UK[‡].
Social media	–	83%	5.66%	5.66%	5.66%	–	–	Based on a survey carried out by Broadband Search about the use of mobile device and desktop or laptop computers for Internet access[§].
Video streaming	720p	15.5%	8%	7%	7%	12.5%	–	Based on a survey of global online video viewers carried out in 2019[**]. The resolution of video streaming was assumed equally distributed between 720p and 1080p.
	1080p	15.5%	8%	7%	7%	–	12.5%	
Music streaming	Standard quality	59%	13.66%	13.66%	13.66%	–	–	Based on a global study held in 2019 about the Internet users' preference for music listening[††]
Video conferencing	Standard quality	–	–	50%	50%	–	–	We assume that video conferencing is equally distributed between laptop and desktop computer due to the lack of data.

‡ Statista: Which one of these devices do you use most for surfing or browsing the internet?

§ Broadband Search: Mobile Vs. Desktop Internet Usage (Latest 2020 Data)

** Statista: Devices used to watch online video worldwide as of August 2019

†† Statista: Share of time spent listening to music on selected devices worldwide as of May 2019

Supplementary Table 4. Electricity intensity of end-user devices, customer premise equipment (CPE), data transmission networks, and data centres. The electricity intensity of data centres and core network is considered proportional to the load and, consequently, is expressed as amount of electricity per unit of data. End-user devices, CPE, and access network are considered agnostic to data load and their electricity intensity is expressed as amount of electricity consumed per active hour. TD: triangular distribution represented by the mean, minimum, and maximum values in brackets.

	Baseline (2050)	Distribution	Units	Assumptions and data source
Smartphone	0.0015 (0.0006)	TD(0.0015, 0.0011, 0.0018)	kWh active $hour^{-1}$	Electricity consumption of a smartphone in 2020 and 2050 was extrapolated from 2016 data (average of 3.34 kWh $year^{-1}$, ranging from 2.519 to 4.161 kWh $year^{-1}$)[7] considering an annual energy usage improvement of 3%[7]. Yearly consumption was converted to consumption per active hour considering 5h 30min of active mode based on a survey prepared by Statista in 2021[‡‡].
Tablet	0.0054 (0.002)	TD(0.0054, 0.0037, 0.0063)	kWh active $hour^{-1}$	Electricity consumption of a tablet in 2020 and 2050 was extrapolated from 2013 data (average of 6.1 kWh $year^{-1}$, ranging from 4.2 kWh $year^{-1}$ for a small tablet to 7.2 kWh $year^{-1}$ for a large tablet)[8] and considering an annual energy usage improvement of 3%[7]. Yearly consumption was converted to consumption per active hour considering 2h and 30min of active mode per day[8]
Laptop	0.0242 (0.0061)	TD(0.0242, 0.0169, 0.0315)	kWh active $hour^{-1}$	Electricity consumption of a laptop in 2020 and 2050 was extrapolated from 2012 data (average of 49 kWh $year^{-1}$)[9] and considering an annual energy usage improvement of 4%[7]. Yearly consumption was converted to consumption per active hour considering 4h of active mode per day[7]. ±30% around the average value was used to define the uncertainty range[7]
Desktop computer	0.1096 (0.0439)	TD(0.1096, 0.0767, 0.1425)	kWh active $hour^{-1}$	Electricity consumption of a desktop computer with LCD monitor in 2020 and 2050 was extrapolated from 2012 data (average of 245 kWh $year^{-1}$)[9] and considering an annual energy usage improvement of 1%[7]. Yearly consumption was converted to consumption per active hour considering 4h 48min of active mode per day[10]. ±30% around the average value was used to define the uncertainty range[7]

‡‡ Statista: How much time on average do you spend on your phone on a daily basis? (2021)

TV 720p	0.0255 (0.0061)	TD(0.0255, 0.0150, 0.0650)	kWh active hour^{-1}	Electricity consumption based on average market data for year 2022[§§]. Electricity used in standby not included (negligible). Extrapolation to 2050 considering an annual energy usage improvement of 5%[11]
TV 1080p	0.0333 (0.0079)	TD(0.033, 0.0145, 0.0850)	kWh active hour^{-1}	Electricity consumption based on average market data for year 2022. Electricity used in standby not included (negligible). Extrapolation to 2050 considering an annual energy usage improvement of 5%[11]
TV 4K	0.0800 (0.0190)	TD(0.0800, 0.0475, 0.1136)	kWh active hour^{-1}	Electricity consumption based on average market data for year 2022. Electricity used in standby not included (negligible). Extrapolation to 2050 considering an annual energy usage improvement of 5%[11]
CPE	0.0070 (0.0038)	TD(0.0070, 0.0040, 0.0100)	kWh active hour^{-1}	Modems and WiFi routers used to access the Internet at home consumes between 4 and 10 Wh[12]. An average value of 7 Wh was assumed as baseline. Extrapolation to 2050 considering an annual energy usage improvement of 2%[11]
Access network	0.0028 (0.0015)	TD(0.0028, 0.0022, 0.0034)	kWh active hour^{-1}	The access network refers to the equipment connecting users to Internet Service Provider (ISP). This equipment consumes about 2.8 Wh[12]. ±20% around the average value was used to define the uncertainty range. Extrapolation to 2050 considering an annual energy usage improvement of 2%[11]
Core network	0.0177 (0.0097)	TD(0.0177, 0.0128, 0.0204)	kWh gigabyte^{-1}	The Internet core network includes mainly routers and fiber optic equipment. The core network consumed 0.02 kWh GB^{-1} in 2014 (25th and 75th percentiles from a Monte Carlo simulation were used to define the uncertainty range)[13]. In order to extrapolate these values to 2020 and 2050, we assumed an annual energy usage improvement of 2%[11]. This improvement can be considered as a conservative assumption as previous works used values as high as 12.5%[13] or 20%[14]
Data centres	0.0414 (0.0266)	TD(0.0414, 0.0310, 0.0517)	kWh gigabyte^{-1}	Electricity consumption of global data centres in 2020 equal to 196.2 TWh[15]. Electricity intensity was calculated by dividing global electricity consumption by the outbound data traffic. According to projections developed by Cisco, only 28% of all

[§§] Ecocostsavings: TV Wattage – 2022'S Most Efficient TVs Revealed [With Data]

	data centres data traffic in 2020 (17.1 ZB) goes to other data centres and users, while 72% is data traffic within the data centres[16]. This gives a reference value of 4.74 ZB outbound traffic in 2020. The lower and upper bounds equal ±25% around the average value, based on the 95% confidence interval obtained by Koot and Wijnhoven[17]. Extrapolation to 2050 considering an annual energy usage improvement of 2%[11]

Supplementary Table 5. Unit of infrastructure allocated per active hour over end-user devices and customer premise equipment (CPE) operating lifetime. TD: triangular distribution represented by the mean, minimum, and maximum values in brackets.

	Lifetime (years)	Active mode (active hours day^{-1})	Infrastructure requirement (unit active hour^{-1})	Assumptions and data source
Smartphone	3	TD(5h 30min, 3h 30min, 7h 00min)	TD(1.66E-04, 1.22E−04, 2.61E−04)	Operating lifetime from Clément *et al.*[18] and active mode per day based on a survey prepared by Statista in 2021 for the U.S.[***]
Tablet	3	TD(2h 30min, 1h 30min, 3h 30min)	TD(3.65E−04, 2.61E−04, 6.08E−04)	Operating lifetime from Clément *et al.*[18] and active mode per day from Urban *et al.*[10] (assuming ±1 hour around the average value to define the uncertainty range)
Laptop	4	TD(4h 00min, 3h 24min, 4h 36min)	TD(1.71E−04, 1.49E−04, 2.01E−04)	Operating lifetime from Malmodin and Lundén[19] and active mode per day from Urban *et al.*[10]
Desktop computer	5	TD(4h 48min, 4h 06min, 4h 30min)	TD(1.14E-04, 9.96E-05, 1.34E-04)	Operating lifetime from Malmodin and Lundén[19] and active mode per day from Urban *et al.*[10]
TV (720p, 1080p, 4K)	10	TD(2h 46min, 2h 00min, 3h 00min)	TD(9.90E-05, 9.13E-05, 1.37E-04)	Operating lifetime consistent with the Ecoinvent 3.8 database[3] and active mode per day based on a survey prepared by Statista in 2020[†††] (assuming 2 and 3 hours to define the uncertainty range)
CPE	5	24h 00min	2.28E-05	Operating lifetime from Ruiz *et al.*[20]. The CPE is normally always on and ready to use since people in general do not turn off the modem/routers[10]. Therefore, we considered 24 hours per day of active mode

[***] Statista: How much time on average do you spend on your phone on a daily basis? (2021)
[†††] Statista: Daily time spent watching TV worldwide from 2011 to 2021

Supplementary Table 6. Unit of data centre infrastructure allocated per gigabyte (GB) of data transferred over the equipment lifetime. To calculate the unit of infrastructure required per GB, we divided the total infrastructure value by the global data centres outbound traffic in 2020 (4.74 zettabytes; see data centres in **Supplementary Table 4**). TD: triangular distribution represented by the mean, minimum, and maximum values in brackets.

	Stock in global data centres (units)	Lifetime (years)	Infrastructure requirement (unit gigabyte^{-1})	Assumptions and data source
Servers	47,556,816	TD(5.5, 3, 10)	TD(1.82E-06, 1.00E-06, 3.34E-06)	Stock of servers in global data centres in 2020 based on Masanet *et al.*[15] and operating lifetime from Fuchs *et al.*[21].
Storage	178,700,000	TD(5.5, 3, 10)	TD(6.85E-06, 3.77E-06, 1.26E-05)	Masanet *et al.*[15] estimated that the storage capacity installed in global data centres in 2020 equals 1,787 EB. This capacity is distributed between solid state drives (31%) and hard disk drives (69%). However, since the Ecoinvent database only provides an inventory for hard disk drives, we consider that 100% of the capacity is of this type. We estimated the stock in global data centres from the total capacity (1,787 EB) and an average specific capacity of 10 TB/hard disk drive[15].
Rack*	1,132,305	TD(5.5, 3, 10)	TD(5.43E-06, 2.99E-06, 9.95E-06)	To calculate the stock of racks in global data centres, we assume 1U servers and 42U, meaning that each rack can hold 42 servers[‡‡‡]. The same lifetime as for servers was assumed. An average weight of 125 kg was assumed for a 42U rack[§§§].

* Infrastructure requirement expressed in kg of rack per GB of data transferred

[‡‡‡] RackSolutions: How many servers does a data center have?
[§§§] 42U: APC 42U Server Racks

Supplementary Table 7. Data traffic demand of digital content. TD: triangular distribution represented by the mean, minimum, and maximum values in brackets.

Digital content	Baseline	Distribution	Units	Assumptions and data source
Web surfing	0.105	TD(0.105, 0.060, 0.150)	gigabyte hour^{-1}	Data obtained from different sources[****]
Social media	0.309	TD(0.309, 0.090, 0.840)	gigabyte hour^{-1}	Data obtained from different sources[††††], [‡‡‡‡], [§§§§]
Video streaming, 480p	0.652	TD(0.652, 0.450, 0.800)	gigabyte hour^{-1}	Data obtained from top video streaming platforms, including YouTube[*****], Netflix[†††††], Amazon Prime[‡‡‡‡‡], Disney+[§§§§§], Hulu[******], and Vimeo[††††††]
Video streaming, 720p	1.267	TD(1.267, 1.000, 1.688)	gigabyte hour^{-1}	
Video streaming, 1080p	2.422	TD(2.422, 1.800, 3.150)	gigabyte hour^{-1}	
Video streaming, 4K	7.624	TD(7.624, 6.000, 9.900)	gigabyte hour^{-1}	
Music streaming, low quality	0.027	TD(0.027, 0.011, 0.043)	gigabyte hour^{-1}	Data obtained from top music streaming platforms, including YouTube Music[‡‡‡‡‡‡] and Spotify[§§§§§§]
Music streaming, standard quality	0.058	TD(0.058, 0.043, 0.072)	gigabyte hour^{-1}	
Music streaming, high quality	0.113	TD(0.113, 0.072, 0.144)	gigabyte hour^{-1}	
Video conferencing, audio only	0.036	TD(0.036, 0.030, 0.045)	gigabyte hour^{-1}	Data obtained from top online meeting platform, including Zoom[*******], Microsoft Teams[†††††††], and Skype[‡‡‡‡‡‡‡]
Video conferencing, standard quality	0.600	TD(0.600, 0.360, 0.840)	gigabyte hour^{-1}	
Video conferencing, high quality	1.384	TD(1.384, 1.238, 1.530)	gigabyte hour^{-1}	

[****] amaysim: Internet Data Usage Guide: What uses most data?
[††††] Wirefly: How Much Data Does The Facebook App Use?
[‡‡‡‡] whatsabyte: How Much Data Does Tik Tok Use Per Hour?
[§§§§] CanstarBlue: How much data does Twitter use?
[*****] YouTube Help: Choose live encoder settings, bitrates, and resolutions
[†††††] Netflix: How to control how much data Netflix uses
[‡‡‡‡‡] MUO: How Much Data Does Streaming Video Use?
[§§§§§] Disney+: Data usage and streaming quality on Disney+
[******] Hulu: Video quality on Hulu
[††††††] vimeo Help Center: Determining playback resolution
[‡‡‡‡‡‡] Smart Home Starter: https://smarthomestarter.com/how-much-data-does-youtube-music-really-use/
[§§§§§§] Spotify: Audio quality
[*******] Zoom Support: Zoom system requirements: Windows, macOS, Linux
[†††††††] Microsoft Teams: Bandwidth requirements
[‡‡‡‡‡‡‡] Skype: How much bandwidth does Skype need?

3 Earth's carrying capacity

This section documents the approach and assumptions to determine the global carrying capacity in each impact category. We define the Earth's carrying capacity in each of the 16 impact categories included in the Environmental Footprint (EF) framework of the European Commission[22]. An overview of the impact categories, impact assessment methods and their quality level and global carrying capacity is presented in **Supplementary Table 8**.

Supplementary Table 8. Overview of the assessed impact categories, impact assessment methods and their quality level, and carrying capacities.

Impact category	Assessment method	Quality level	Carrying capacity	Unit
Climate change	IPCC 2021[1]	I	400	Gt CO_2 over 2020-2100
Acidification	EF 3.0	II	$1{\cdot}10^{12}$	mol H^+-eq year^{-1}
Eutrophication, freshwater	EF 3.0	II	5.81	Mt P-eq year^{-1}
Eutrophication, marine	EF 3.0	II	23	Mt N-eq year^{-1}
Eutrophication, terrestrial	EF 3.0	II	$6.13{\cdot}10^{12}$	mol N-eq year^{-1}
Photochemical ozone formation	EF 3.0	II	407	Mt NMVOC-eq year^{-1}
Particulate matter formation	EF 3.0	I	$7.48{\cdot}10^{-5}$	disease incidence per person per year
Ozone depletion	EF 3.0	I	0.36	Mt CFC-11-eq year^{-1}
Ecotoxicity, freshwater	EF 3.1[23]	II/III	$1.30{\cdot}10^{14}$	CTUe year^{-1}
Human toxicity, carcinogenic	EF 3.1[23]	II/III	$1.39{\cdot}10^{-4}$	CTUh per person per year
Human toxicity, non-carcinogenic	EF 3.1[23]	II/III	$5.93{\cdot}10^{-4}$	CTUh per person per year
Ionising radiation	EF 3.0	II	$7.62{\cdot}10^{4}$	kBq U^{235}-eq per person per year
Resource use, fossils	EF 3.0	III	224	EJ year^{-1}
Resource use, mineral and metals	EF 3.0	III	219	kt Sb-eq year^{-1}
Land use, soil erosion	LANCA v2.5[24]	III	12.7	Gt soil loss year^{-1}
Freshwater use	Inventory entries for water withdrawal	N.A.	4,000	km^3 year^{-1}

Impact assessment methods are classified according to their quality level into: I: recommended and satisfactory, II: recommended but further improvements are needed, and III: recommended but should be applied with caution[25]. CTUe: Comparative Toxic Unit for ecosystems. CTUh: Comparative Toxic Unit for humans. DALY: Disability Adjusted Life Years. NMVOC: non-methane volatile organic compounds.

Climate change

The climate change impact category captures the warming potential of the climate system due to greenhouse gas (GHG) emissions linked to human activity (i.e., anthropogenic GHG emissions). To define a carrying capacity for climate change, we follow the Paris Agreement goal of limiting global warming to well below 2 °C, preferably to 1.5 °C, above the pre-industrial level. Following the precautionary principle adopted in the planetary boundary framework[26,27], we choose the 1.5 °C limit as well as a stringent carbon budget that would allow reaching this target with a high likelihood. Hence, we define the carrying capacity base on the Intergovernmental Panel on Climate Change (IPCC) Sixth Assessment Report (AR6) estimates of the remaining

carbon budget from the beginning of 2020 until global net zero CO_2 emissions are reached with a high probability (67%) of limiting global warming to 1.5 °C by 2100[1]. This carbon budget corresponds to the cumulative emission of 400 Gt CO_2 over the period 2020-2100. Moreover, the influence of different carbon budgets corresponding to different temperature limit targets and likelihoods of reaching these targets is tested in **Section 1.1**.

Acidification

The acidification category quantifies the impacts on terrestrial ecosystems due to the emission of acidifying substances (e.g., NO_x, NH_3 and SO_2). Björn and Hauschild[28] proposed a threshold for terrestrial acidification based on the critical load concept, i.e., "*the highest deposition of acidifying compounds that will not cause chemical changes leading to long-term harmful effects on ecosystem structure and function*". They estimated an average global critical load of 1,170 mol H^+-eq per ha per year, from which natural depositions (90 mol H^+-eq per ha per year) have to be subtracted. Based on the final threshold (1,080 mol H^+-eq per ha per year) and using the global terrestrial area ($1.49 \cdot 10^{10}$ ha), the authors derived a global carrying capacity of $1.59 \cdot 10^{13}$ mol H^+-eq year^{-1}. This carrying capacity was further refined in Sala *et al.*[29] in order to adapt it to the EF's method for acidification, resulting in a new global carrying capacity of $1 \cdot 10^{12}$ mol H^+-eq year^{-1}. Other works have estimated the acidification's threshold and carrying capacity. For example, Björn *et al.*[30] found a threshold ranging from 100 to 4,000 mol H^+-eq per ha per year, with a median value of 500 mol H^+-eq per ha per year. Based on the median value and considering the global ice-free land area ($1.21 \cdot 10^{10}$ ha), Gebara and Laurent[31] derived a global carrying capacity of $6.05 \cdot 10^{12}$ mol H^+-eq year^{-1}. We consider the carrying capacity as calculated in Sala *et al* in order to be consistent with the EF method.

Eutrophication, freshwater

The freshwater eutrophication impact category quantifies the fraction of phosphorus (P) compounds released to water and soil that reaches freshwater ecosystems. Björn and Hauschild[28] derived a carrying capacity based on a generic threshold of 0.3 mg P_{tot} L^{-1} as "*concentrations above this value are considered a potential cause of encroachment of aquatic life due to nutrient enrichment*". The authors translated this concentration threshold to a carrying capacity by linking a marginal emission increase to a steady state concentration increase and subtracting the natural background level. The reported global carrying capacity is 5.81 Mt P-eq year^{-1}.

Eutrophication, marine

The marine eutrophication impact category quantifies the fraction of nitrogen (N) compounds released to water (e.g., N and NO_3^-) and air (e.g., NO_x and NH_3) that reaches marine ecosystems. Gebara and Laurent[31] estimated a global carrying capacity for marine eutrophication equal to 23 Mt N-eq year^{-1}. They used a spatially-resolved dataset of O_2 concentration for the reference state and the limit across the 66 large marine ecosystems[32] to quantify the annual N emissions to sea that would allow staying below the concentration threshold.

Eutrophication, terrestrial

As for terrestrial acidification, Björn and Hauschild[28] proposed a threshold for terrestrial eutrophication based on the critical load concept, which here is defined as "*the highest deposition of nitrogen as NH_x and/or NO_y below which harmful effects in ecosystem structure and function do not occur according to present knowledge*". The global critical load was estimated at 1,340 mol N-eq per ha per year after subtracting natural depositions. Based on this threshold and using the global terrestrial area ($1.49 \cdot 10^{10}$ ha), the authors derived a global carrying capacity of $1.94 \cdot 10^{12}$ mol N-eq year^{-1}. This carrying capacity was further refined in Sala *et al.*[29] in order to adapt it to the EF's method for terrestrial eutrophication, resulting in a new global carrying capacity

of $6.13 \cdot 10^{12}$ mol N-eq year^{-1}. In the current work, we consider the carrying capacity as calculated in Sala *et al.* in order to be consistent with the EF method.

Photochemical ozone formation

Ozone (O_3) has been recognized as a potential risk for human health. The photochemical ozone formation impact category quantifies the increase in tropospheric ozone concentration due to the emission of precursors (e.g., NO_x, CO, and volatile organic compounds (VOC)) that can react in the presence of UV light to form O_3. Björn and Hauschild[28] derived a global carrying capacity for photochemical ozone formation based on an ozone concentration threshold of 3 ppm per hour AOT40 for daylight hours during May-July (AOT40 is an "*effect measure calculated as the accumulated ozone exposure during daylight hours above a concentration of 40 ppb*"). The authors translated the concentration threshold into annual emissions of non-methane VOC (NMVOC) by applying the average European fate factor for ozone ($5.8 \cdot 10^{-14}$ kg O_3 per m3 per kg NMOVC per day) and the global land area. The calculated carrying capacity equals 26 Mt NMVOC-eq year^{-1}. However, in a subsequent work, Vargas-Gonzalez *et al.*[33] argued that the used fate factor for ozone is outdated. Thus, the authors performed the calculations using a new fate factor ($2.4 \cdot 10^{-15}$ kg O_3 per m3 per kg NMOVC per day) and resulting in a substantially higher carrying capacity of 407 Mt NMVOC-eq year^{-1}, which was also adopted in the work of Sala *et al.*[29] and in the current work.

Particulate matter formation

Particulate matter (PM) represents a major risk for human health. The impact category quantifies the disease incidence due to PM formation as both direct PM emissions (including particles with diameter <2.5 µg, between 2.5 and 10 µg, and >10 µg) and secondary PM formed from NO_x, NH_3 and SO_X emissions. The World Health Organization (WHO)[34] recommends annual average PM concentration levels below 10 µg of $PM_{2.5}$ m^{-3}. Based on this concentration threshold and considering the average breathing rate (13 m^3 per person per day) and human health burden of $PM_{2.5}$ (78 Disability Adjusted Life Years (DALY) kg^{-1}), Vargas-Gonzalez *et al.*[33] derived an acceptable environmental burden of 0.0016 DALY per person per year. The method recommended in the EF for the assessment of particulate matter formation quantifies disease incidence instead of DALY. Hence, we apply the corresponding conversion factor (21.4 DALY disease incidence^{-1})[29] to arrive at a global threshold of $7.48 \cdot 10^{-5}$ disease incidence per person per year.

Ozone depletion

The depletion of stratospheric ozone results in less absorption of solar radiation and an increased UV radiation at the Earth's surface. The impact category quantifies the ozone depletion potential. The planetary boundary for ozone depletion has been proposed in Steffen *et al.*[27] at 5% reduction of stratospheric ozone concentration compared to the pre-industrial level, with an uncertainty range from 5% to 10%. Björn and Hauschild[28] translated the average concentration threshold to a carrying capacity expressed in kg CFC-11-eq year^{-1} based on a model that calculates the sustained CFC-11-eq emissions that would result in a 7.5% decrease in ozone levels at steady state. In a subsequent work, Gebara and Laurent[31] applied the same model to the entire uncertainty range, resulting in a carrying capacity ranging from 360 kt CFC-11-eq year^{-1} for a 5% reduction to 720 kt CFC-11-eq year^{-1} for a 10% reduction. Here we adopt the carrying capacity for the lower bound of 5% following the planetary boundaries framework[27].

Ecotoxicity, freshwater

Björn and Hauschild[28] derived a carrying capacity for freshwater ecotoxicity based on the threshold HC5(NOEC), which is defined as "*the concentration at which maximum 5% of species in an ecosystem are affected*". The authors calculated a global carrying capacity of $1.30 \cdot 10^{14}$ Comparative Toxic Unit for ecosystems

(CTUe) year^{-1}, which has been also adopted in Sala *et al.*[29] and in the current work. Moreover, we use the updated method available in EF 3.1[23] to evaluate the freshwater ecotoxicity impact.

Human toxicity (carcinogenic and non-carcinogenic) and ionising radiation

A threshold for human toxicity and ionising radiation has been proposed in Vargas-Gonzalez *et al.*[33] based on the acceptable environmental burden also applied to particulate matter formation (i.e., 0.0016 DALY per person per year). The authors propose the same acceptable burden across all the impact categories related to human health in order to have a consistent approach. The conversion factors to EF units are 11.5 DALY Comparative Toxic Unit for humans^{-1} (CTUh) for carcinogenic human toxicity, 2.7 DALY CTUh^{-1} for non-carcinogenic human toxicity, and $2.10\cdot10^{-8}$ DALY kBq U^{235}-eq^{-1} for ionising radiation[29]. Just as for ecotoxicity, we use the updated method available in EF 3.1[23] to evaluate the human toxicity impacts.

Resource use (fossils and mineral and metals)

In contrast to the previous impact categories, defining a physical threshold for resource use is rather challenging as these indicators typically quantify the depletion of valuable materials[33]. Several attempts have been made to estimate resource budgets. For example, Sala *et al.*[29] followed a more normative approach by applying the concept of Factor 2[35,36], according to which global resource use needs to be reduced by 50% to achieve environmental sustainability. Accordingly, they estimate that the global thresholds should be 224 EJ year^{-1} for fossil resource use and 219 kt Sb-eq year^{-1} for mineral and metal resources depletion. Vargas-Gonzalez *et al.*[33] proposed the optimal extraction rate, which is the ration between the available stock of a given resource and the number of years necessary to adapt to the lack of that resource. Based on this approach, they estimate that the resource depletion reduction factor should be 4.08. Gebara and Laurent[31] further applied the optimal extraction rate concept to estimate an aggregated global threshold for oil, coal, and natural gas of 5.7 Gtoe year^{-1} or 239 EJ year^{-1}, very similar to the value proposed by Sala *et al.* Moreover, Desing *et al.*[37] proposed a method to estimate resource budgets based on the hypothesis that resources are not limited by their availability but rather by the environmental impacts caused. In this way, the authors estimate that the budget for major metals that would keep the sector within its share of the Earth's system boundaries is 40 times smaller than production volumes in 2016.

In the current work, we use the resource budgets derived by Sala *et al.*[29] based on the Factor 2 concept. This is done in order to be consistent with the EF methods for fossil and mineral and metal resources use. This assumption results in a lower bound for the carrying capacity share consumed by digital content consumption, since the other studies suggest more stringent budgets.

Land use, soil erosion

The land use impact category evaluated in this study captures the impact on soil erosion due to land occupation and transformation. This approach is preferred over a more simplistic land accounting method (i.e., quantifying the amount of land occupied and/or transformed over the life cycle of a product or service) since it considers changes in soil properties and functions[38]. Björn and Hauschild[28] defined the threshold based on the tolerable soil erosion, defined as the "*soil erosion rate at which a deterioration or loss of one or more soil functions does not occur*". This threshold is equal to 0.85 t per ha per year on average, with an interval ranging from 0.3 to 1.4 t per ha per year. The authors multiplied the average soil erosion threshold with the global terrestrial area ($1.49\cdot10^{10}$ ha), resulting in a global carrying capacity of 12.7Gt soil loss year^{-1}. To quantify the land use impacts, we use the characterization factors for soil erosion due to land occupation and transformation as provided in the LANCA v2.5 model[24].

Freshwater use

The freshwater use category quantifies water withdrawals, i.e., volume of freshwater taken from ground and/or surface water sources. The planetary boundary for freshwater use has been proposed in Rockström *et al.*[26] at 4,000 km^3 $year^{-1}$ (uncertainty range of 4,000-6,000 km^3 $year^{-1}$), which refers to the maximum consumptive use of blue water.

4 Methodological assumptions, limitations, and future work

Here we extend the discussion presented in the main manuscript around the major assumptions and limitations of our study and their implications, and provide an outline of potential future research directions.

- Our analysis focuses on the environmental impacts of digital content consumption, including web surfing, social media, video and music streaming, and video conferencing. It is worth mentioning that the Internet plays a role in many other aspects of our daily life. For example, the Cisco's estimates show that there are around 30 billion connected devices[41], most of which are Internet of Things (IoT) devices such as various kinds of sensors used for automation in smart homes. Online gaming is another example of activity not considered in our analysis. Addressing these other activities that rely on Internet connection possesses serious challenges, particularly in terms of data availability (e.g., one would need to know the daily activity of a range of gadgets used in smart homes). The five digital services assessed in our work encompass the primary reasons for Internet usage among users[6]. Furthermore, the inclusion of additional activities would not alter the insights derived from our study, as it would further increase the impacts.
- The definition of user's consumption patterns requires specific information about the annual number of hours spent on each digital content as well as the user's preference regarding quality/resolution settings and the devices used to access digital content. It is worth noting that this type of information is scarce and not commonly found in official reports or peer-reviewed papers. Hence, we obtained this data from the grey literature and online platforms specialized in market and consumer data, such as Statista (as documented in Supporting Section 2). We addressed the issue of variability in user's consumption pattern by exploring two alternative user archetypes that differ in their preferences (e.g., regarding the type of device used). These results show that users' preferences can largely influence the environmental outcome (**Supplementary Fig. 6** and **Supplementary Fig. 7**). Improving the availability of data on user consumption patterns would enable more comprehensive studies, including, e.g., LCAs that consider the geographical variation of digital content consumption patterns.
- The system boundaries are defined as cradle-to-grave, thereby including all the relevant impacts throughout the manufacturing, distribution, operation, and end-of-life management of each system's component. Nevertheless, we omit the infrastructure of the data transmission network (e.g., routers and undersea cables) due to the lack of data and the substantially higher environmental importance of the use stage[19,42,43]. Moreover, we omit the energy required for the creation and upload of online content (e.g., videos)[44,45]. These are, arguably, minor elements of the Internet infrastructure and that their impacts are deemed negligible compared with, e.g., the manufacture and operation of end-user devices and data centres.
- The consumption-based perspective used in this work implies that the impacts generated throughout the life cycle of Internet provision are assigned to the user who consumes digital services. It is worth mentioning that some digital services may be shared among multiple users. For example, two people can watch video streaming together on a TV. Ideally, the impacts should be distributed among the two people. However, implementing such allocation procedures would require additional information about consumption patterns, such as the proportion of time an average user watches video streaming alone or with others. In light of this, we chose to allocate all impacts to the average user, which is equivalent to assuming that the user does not share digital services with others.
- We follow common recommendations in the literature to model electricity usage during the operation of the Internet infrastructure[46]. Accordingly, we considered that the electricity intensity of data centres and the core network is proportional to data load, while the electricity intensity of end-user devices, CPE, and

access network is proportional to the active hours. We acknowledge that the way of measuring the energy consumption of network devices is subject to debate, and could be a source of uncertainty[46,47].

- We provide the environmental impacts of digital content consumption for a range of user locations (**Figure 2** in the main manuscript). To obtain these results, we adapted the electricity mix that powers electronic devices to the user location. Most of the other inventories remain unchanged across countries. For example, the same inventories were considered for data centres or electronic devices manufacturing, regardless of the user location. This assumption is based on the fact that these inventories represent products/services that are sourced from global markets. For example, data centres are spread all over the globe, while electronic devices manufacturing primarily takes place in China to be distributed worldwide.
- We used 2020 as the reference year for LCI data whenever possible. If the electricity intensity of electronic devices was not available for 2020, we extrapolated the most recent values by applying an annual energy usage improvement rate based on the literature. For the manufacturing of electronic devices, we have retrieved the corresponding LCI data from the ecoinvent database v3.8. We acknowledge that some of the data in the ecoinvent database may not capture the latest developments due to the rapid pace of change in the manufacturing industry. For example, the LCI data for the production of a smartphone included in the latest version of ecoinvent is based on a 2014 LCA report. We assessed the robustness of our conclusions under uncertainties in the LCI data via the Monte Carlo sampling method (Error! Reference s ource not found.), finding that despite the large uncertainty in some impact categories, the general outcome of our analysis likely remains largely valid.
- We considered that the electricity required by data centres is supplied by an average electricity mix weighted according to the share of data centres in each region. We acknowledge that ICT companies are procuring an increasing amount of renewable electricity, leading to potential reductions in operational emissions and an increased contribution of embodied emissions to the overall environmental footprint. In 2021, Apple, Google, and Meta acquired or generated renewable electricity equivalent to 100% of their operational electricity demand[48]. However, it is important to note that this does not mean that their data centres are supplied exclusively by renewable electricity, largely due to the variability of renewable energies[48,49]. Moreover, not all ICT companies are transparent in reporting disaggregated electricity consumption and sources[50], making it very challenging to accurately determine the proportion of global data traffic attributed to data centres powered by renewable electricity. We conducted a sensitivity analysis to explore the impact of an increased renewable electricity procurement by data centres. We found that if global data centres were entirely powered by wind or solar PV, the most critical impacts would be reduced by ca. 30% for climate change, 24% for freshwater eutrophication, 15% for marine eutrophication, 21% for particulate matter formation, 11% for freshwater ecotoxicity, and <2% for mineral and metal resources depletion (**Supplementary Fig. 4**).
- We computed the annual per capita carbon budget following the approach proposed by Dao *et al.*[39]. Hence, we divided the cumulative carbon budget over the period 2020-2100 by the cumulative global population over the same period. Other studies have distributed the cumulative carbon budget over a shorter timeframe, typically until 2050 under the assumption that net-zero emissions would be achieved by then[51]. A shorter timeframe would result in a higher annual per capita carbon budget, since the same cumulative carbon budget is distributed over a less years. However, we argue that a timeframe until 2100 is a reasonable and commonly used approach which aligns with IPCC scenarios[39,52,53].
- We omit the time dimension when quantifying emissions and environmental impacts. Notably, previous studies have demonstrated that users primarily watch video streaming in the evenings/nights and on weekends[44]. This pattern could affect emissions and impacts, as the electricity generation mix during these times may differ from the annual average mix used in our analysis. Future research could focus on

analysing video streaming at an hourly resolution, in combination with hourly electricity generation data, to provide more accurate estimates.

- The environmental consequences of further Internet expansion in the next decades will depend on other factors omitted in this study. Notably, per capita demand for digital content could further increase due to rebound effects, i.e., cheaper or more performant Internet access might result in higher data traffic[54]. Such effects were omitted due to lack of data, yet we acknowledge their potential role in a study of this type.

5 Supplementary references

1. IPCC. *Climate Change 2021: The Physical Science Basis*. https://www.ipcc.ch/report/ar6/wg1/ (2021).
2. Stehfest, E., van Vuuren, D., Kram, T. & Bouwman, L. *Integrated Assessment of Global Environmental Change with IMAGE 3.0: Model description and policy applications*. (2014).
3. Wernet, G. *et al.* The ecoinvent database version 3 (part I): overview and methodology. *Int. J. Life Cycle Assess.* **21**, 1218–1230 (2016).
4. Pizzol, M. Deterministic and stochastic carbon footprint of intermodal ferry and truck freight transport across Scandinavian routes. *Journal of Cleaner Production* **224**, 626–636 (2019).
5. Istrate, I.-R. Dataset to calculate the environmental footprint of digital content consumption. (2023) doi:10.5281/zenodo.7575568.
6. Hootsuite. *Digital 2022 - Global overview report - The essential guide to the World's connected behaviour*. https://datareportal.com/reports/digital-2022-global-overview-report (2022).
7. Pärssinen, M., Kotila, M., Cuevas, R., Phansalkar, A. & Manner, J. Environmental impact assessment of online advertising. *Environmental Impact Assessment Review* **73**, 177–200 (2018).
8. Urban, B., Shmakova, V., Lim, B. & Roth, K. *Energy Consumption of Consumer Electronics in U.S. Homes in 2013*. (2014).
9. Van Heddeghem, W. *et al.* Trends in worldwide ICT electricity consumption from 2007 to 2012. *Computer Communications* **50**, 64–76 (2014).
10. Urban, B., Roth, K., Singh, M. & Howes, D. *Energy Consumption of Consumer Electronics in. U.S. Homes in 2017*. (2017).
11. Madlener, R., Sheykhha, S. & Briglauer, W. The electricity- and CO2-saving potentials offered by regulation of European video-streaming services. *Energy Policy* **161**, 112716 (2022).
12. Coroama, V. C., Schien, D., Preist, C. & Hilty, L. M. The Energy Intensity of the Internet: Home and Access Networks. in *ICT Innovations for Sustainability* (eds. Hilty, L. M. & Aebischer, B.) vol. 310 137–155 (Springer International Publishing, 2015).

13. Schien, D. & Preist, C. Approaches to energy intensity of the internet. *IEEE Communications Magazine* **52**, 130–137 (2014).
14. Andrae, A. S. G. New perspectives on internet electricity use in 2030. *Engineering and Applied Science Letters* **3**, 19–31 (2020).
15. Masanet, E., Shehabi, A., Lei, N., Smith, S. & Koomey, J. Recalibrating global data center energy-use estimates. *Science* **367**, 984–986 (2020).
16. Cisco. *Cisco Global Cloud Index: Forecast and Methodology, 2016–2021*. https://virtualization.network/Resources/Whitepapers/0b75cf2e-0c53-4891-918e-b542a5d364c5_white-paper-c11-738085.pdf (2018).
17. Koot, M. & Wijnhoven, F. Usage impact on data center electricity needs: A system dynamic forecasting model. *Applied Energy* **291**, 116798 (2021).
18. Clément, L.-P. P.-V. P., Jacquemotte, Q. E. S. & Hilty, L. M. Sources of variation in life cycle assessments of smartphones and tablet computers. *Environmental Impact Assessment Review* **84**, 106416 (2020).
19. Malmodin, J. & Lundén, D. The Energy and Carbon Footprint of the Global ICT and E&M Sectors 2010–2015. *Sustainability* **10**, 3027 (2018).
20. Ruiz, D. *et al.* Life cycle inventory and carbon footprint assessment of wireless ICT networks for six demographic areas. *Resources, Conservation and Recycling* **176**, 105951 (2022).
21. Fuchs, H. *et al. Characteristics and Energy Use of Volume Servers in the United States*. https://escholarship.org/uc/item/8bb5j7ww (2017).
22. European Commission. *Commission Recommendation of 16.12.2021 on the use of the Environmental Footprint methods to measure and communicate the life cycle environmental performance of products and organisations*. https://eur-lex.europa.eu/resource.html?uri=cellar:75e0de0f-5e6d-11ec-9c6c-01aa75ed71a1.0019.02/DOC_1&format=PDF (2021).
23. Sala, S., Biganzoli, F., Mengual, E. S. & Saouter, E. Toxicity impacts in the environmental footprint method: calculation principles. *Int J Life Cycle Assess* **27**, 587–602 (2022).

24. Bos, U., Horn, R., Beck, T., Lindner, J. P. & Fischer, M. LANCA - Characterization Factors for Life Cycle Impact Assessment, Version 2.5. *Fraunhofer Verlag* https://www.bookshop.fraunhofer.de/buch/LANCA/244600 (2018).
25. Fazio, S. *et al. Supporting information to the characterisation factors of recommended EF Life Cycle Impact Assessment methods: new methods and differences with ILCD.* (2018).
26. Rockström, J. *et al.* A safe operating space for humanity. *Nature* **461**, 472–475 (2009).
27. Steffen, W. *et al.* Planetary boundaries: Guiding human development on a changing planet. *Science* **347**, 1259855 (2015).
28. Bjørn, A. & Hauschild, M. Z. Introducing carrying capacity-based normalisation in LCA: framework and development of references at midpoint level. *Int J Life Cycle Assess* **20**, 1005–1018 (2015).
29. Sala, S., Crenna, E., Secchi, M. & Sanyé-Mengual, E. Environmental sustainability of European production and consumption assessed against planetary boundaries. *Journal of Environmental Management* **269**, 110686 (2020).
30. Bjørn, A., Margni, M., Roy, P.-O., Bulle, C. & Hauschild, M. Z. A proposal to measure absolute environmental sustainability in life cycle assessment. *Ecological Indicators* **63**, 1–13 (2016).
31. Gebara, C. H. & Laurent, A. National SDG-7 performance assessment to support achieving sustainable energy for all within planetary limits. *Renewable and Sustainable Energy Reviews* **173**, 112934 (2023).
32. Bjørn, A. *et al.* A comprehensive planetary boundary-based method for the nitrogen cycle in life cycle assessment: Development and application to a tomato production case study. *Science of The Total Environment* **715**, 136813 (2020).
33. Vargas-Gonzalez, M. *et al.* Operational Life Cycle Impact Assessment weighting factors based on Planetary Boundaries: Applied to cosmetic products. *Ecological Indicators* **107**, 105498 (2019).
34. World Health Organization. Occupational and Environmental Health Team. *WHO Air quality guidelines for particulate matter, ozone, nitrogen dioxide and sulfur dioxide: global update 2005: summary of risk assessment*. https://apps.who.int/iris/handle/10665/69477 (2006).

35. Buczko, C., Hinterberger, F. & Stricks, V. Towards SDG implementation: The role of global resource policy and resource targets. (2016).

36. Bringezu, S. Possible Target Corridor for Sustainable Use of Global Material Resources. *Resources* **4**, 25–54 (2015).

37. Desing, H., Braun, G. & Hischier, R. Ecological resource availability: a method to estimate resource budgets for a sustainable economy. *Global Sustainability* **3**, e31 (2020).

38. De Laurentiis, V. *et al.* Soil quality index: Exploring options for a comprehensive assessment of land use impacts in LCA. *Journal of Cleaner Production* **215**, 63–74 (2019).

39. Dao, H., Peduzzi, P. & Friot, D. National environmental limits and footprints based on the Planetary Boundaries framework: The case of Switzerland. *Global Environmental Change* **52**, 49–57 (2018).

40. United Nations. World Population Prospects - Population Division - United Nations. https://population.un.org/wpp/ (2022).

41. Cisco. *Cisco Annual Internet Report - Cisco Annual Internet Report (2018–2023) White Paper - Cisco*. https://www.cisco.com/c/en/us/solutions/collateral/executive-perspectives/annual-internet-report/white-paper-c11-741490.html (2020).

42. Tao, Y., Steckel, D., Klemeš, J. J. & You, F. Trend towards virtual and hybrid conferences may be an effective climate change mitigation strategy. *Nat Commun* **12**, 7324 (2021).

43. Whitehead, B., Andrews, D. & Shah, A. The life cycle assessment of a UK data centre. *Int J Life Cycle Assess* **20**, 332–349 (2015).

44. Suski, P., Pohl, J. & Frick, V. All you can stream: Investigating the role of user behavior for greenhouse gas intensity of video streaming. in *Proceedings of the 7th International Conference on ICT for Sustainability* 128–138 (ACM, 2020). doi:10.1145/3401335.3401709.

45. Preist, C., Schien, D. & Shabajee, P. Evaluating Sustainable Interaction Design of Digital Services: The Case of YouTube. in *Proceedings of the 2019 CHI Conference on Human Factors in Computing Systems* 1–12 (ACM, 2019). doi:10.1145/3290605.3300627.

46. Billstein, T., Björklund, A. & Rydberg, T. Life Cycle Assessment of Network Traffic: A Review of Challenges and Possible Solutions. *Sustainability* **13**, 11155 (2021).
47. Coroama, V. C. & Hilty, L. M. Assessing Internet energy intensity: A review of methods and results. *Environmental Impact Assessment Review* **45**, 63–68 (2014).
48. Kamiya, G. *Data Centres and Data Transmission Networks*. https://www.iea.org/reports/data-centres-and-data-transmission-networks (2022).
49. Siddik, A. B., Shehabi, A. & Marston, L. The environmental footprint of data centers in the United States. *Environ. Res. Lett.* **16**, 064017 (2021).
50. Mytton, D. & Ashtine, M. Sources of data center energy estimates: A comprehensive review. *Joule* **6**, 2032–2056 (2022).
51. Chancel, L. Global carbon inequality over 1990–2019. *Nat Sustain* **5**, 931–938 (2022).
52. O'Neill, D. W., Fanning, A. L., Lamb, W. F. & Steinberger, J. K. A good life for all within planetary boundaries. *Nat Sustain* **1**, 88–95 (2018).
53. Lucas, P. L., Wilting, H. C., Hof, A. F. & van Vuuren, D. P. Allocating planetary boundaries to large economies: Distributional consequences of alternative perspectives on distributive fairness. *Global Environmental Change* **60**, 102017 (2020).
54. Bieser, J., Salieri, B., Hischier, R. & Hilty, L. M. Opportunities of 5G Mobile Technology for Climate Protection in Switzerland. in *Advances and New Trends in Environmental Informatics* (eds. Wohlgemuth, V., Naumann, S., Behrens, G., Arndt, H.-K. & Höb, M.) 217–235 (Springer International Publishing, 2023). doi:10.1007/978-3-031-18311-9_13.
