# Supplementary material for: The environmental sustainability of digital content consumption
Source: Nat Commun. 2024 May 2;15:3724. doi: 10.1038/s41467-024-47621-w (PMC11066053; doi:10.1038/s41467-024-47621-w)
Supplement: Supplementary file 3 — Reporting Summary [file 41467_2024_47621_MOESM3_ESM.pdf]

nature portfolio

Corresponding author(s): Gonzalo Guillén-Gosálbez

Last updated by author(s): Mar 20, 2024

Reporting Summary

Nature Portfolio wishes to improve the reproducibility of the work that we publish. This form provides structure for consistency and transparency in reporting. For further information on Nature Portfolio policies, see our Editorial Policies and the Editorial Policy Checklist.

Statistics

For all statistical analyses, confirm that the following items are present in the figure legend, table legend, main text, or Methods section.

n/a	Confirmed	
☐	☒	The exact sample size (*n*) for each experimental group/condition, given as a discrete number and unit of measurement
☒	☐	A statement on whether measurements were taken from distinct samples or whether the same sample was measured repeatedly
☒	☐	The statistical test(s) used AND whether they are one- or two-sided<br>*Only common tests should be described solely by name; describe more complex techniques in the Methods section.*
☒	☐	A description of all covariates tested
☒	☐	A description of any assumptions or corrections, such as tests of normality and adjustment for multiple comparisons
☐	☒	A full description of the statistical parameters including central tendency (e.g. means) or other basic estimates (e.g. regression coefficient) AND variation (e.g. standard deviation) or associated estimates of uncertainty (e.g. confidence intervals)
☒	☐	For null hypothesis testing, the test statistic (e.g. *F*, *t*, *r*) with confidence intervals, effect sizes, degrees of freedom and *P* value noted<br>*Give P values as exact values whenever suitable.*
☒	☐	For Bayesian analysis, information on the choice of priors and Markov chain Monte Carlo settings
☒	☐	For hierarchical and complex designs, identification of the appropriate level for tests and full reporting of outcomes
☒	☐	Estimates of effect sizes (e.g. Cohen's *d*, Pearson's *r*), indicating how they were calculated

Our web collection on statistics for biologists contains articles on many of the points above.

Software and code

Policy information about availability of computer code

Data collection	No software was used for data collection
Data analysis	The following open source Python-based sotware was used for data analysis: Brightway2 v2.4.4 for LCA modelling and calculations; premise v1.4.2 for prospective LCA modelling; pandas v1.5.3 for descriptive statistics; matplotlib v3.7.1 for visualization. The complete Python code used for the calculation and visualization of the results is available at https://doi.org/10.5281/zenodo.8122381

For manuscripts utilizing custom algorithms or software that are central to the research but not yet described in published literature, software must be made available to editors and reviewers. We strongly encourage code deposition in a community repository (e.g. GitHub). See the Nature Portfolio guidelines for submitting code & software for further information.

Data

Policy information about availability of data

All manuscripts must include a data availability statement. This statement should provide the following information, where applicable:

- Accession codes, unique identifiers, or web links for publicly available datasets
- A description of any restrictions on data availability
- For clinical datasets or third party data, please ensure that the statement adheres to our policy

The raw data used to create the LCIs are provided in the Supplementary Information file. The LCI datasets generated in this study are available at https://

doi.org/10.5281/zenodo.8122381. The background LCI datasets used in this study are available in the ecoinvent v3.8 (cut-off system model) database under accessible link https://ecoinvent.org/.

Research involving human participants, their data, or biological material

Policy information about studies with human participants or human data. See also policy information about sex, gender (identity/presentation), and sexual orientation and race, ethnicity and racism.

Reporting on sex and gender	This information has not been collected.
Reporting on race, ethnicity, or other socially relevant groupings	This information has not been collected.
Population characteristics	This information has not been collected.
Recruitment	This information has not been collected.
Ethics oversight	This information has not been collected.

Note that full information on the approval of the study protocol must also be provided in the manuscript.

Field-specific reporting

Please select the one below that is the best fit for your research. If you are not sure, read the appropriate sections before making your selection.

☐ Life sciences ☐ Behavioural & social sciences ☒ Ecological, evolutionary & environmental sciences

For a reference copy of the document with all sections, see nature.com/documents/nr-reporting-summary-flat.pdf

Ecological, evolutionary & environmental sciences study design

All studies must disclose on these points even when the disclosure is negative.

Study description	This study employs the life cycle assessment methodology to quantify the environmental impact of digital content consumption across 16 indicators related to climate change, nutrients flows, air pollution, toxicity, and resources use. Moreover, it compares these impacts with the per capita share of the Earth's carrying capacity, representing explicit environmental intervention thresholds that should never be surpassed. The analysis focuses on the global average consumption patterns across all Internet users, including web surfing, social media, video streaming, music streaming, and video conferencing. The findings show that the consumption of these digital services could account for approximately 40% of the per capita carbon budget consistent with limiting global warming to 1.5 ° C, as well as around 55% of the per capita carrying capacity for mineral and metal resources use and over 10% for five other impact categories. The study underscores the importance of decarbonising electricity to mitigate the climate impacts of Internet consumption. However, it also highlights that the use of mineral and metal resources would remain of concern. Therefore, the study emphasizes the need for a synergistic combination of rapid decarbonisation and additional measures aimed at reducing the use of fresh raw materials in electronic devices (e.g., lifetime extension) to prevent the growing Internet demand from exacerbating the pressure on the finite Earth's carrying capacity.
Research sample	All data used in this study are from existing datasets. Data on digital consumption was obtained from statistic reports (like Statista). LCI data was obtained from the scientific literature combined with the ecoinvent v3.8 database.
Sampling strategy	The uncertainties linked to the LCA results due to uncertainties in the life cycle inventory (LCI) data was assessed by error propagation via the Monte Carlo sampling method based on 1,000 runs. For each run, LCI data values were randomly sampled according to their probability distribution and the life cycle environmental impacts of digital content consumption for the global average user were quantified. The impact value for each run and category was then compared against the corresponding per capita Earth's carrying capacity. Probability distributions were determined based on the literature for Internet network components data and the ecoinvent database v3.83 for background inventory data (i.e., manufacturing processes, power generation, etc.).
Data collection	All data are from publicly available sources documented in the main manuscript and the Supplementary Information file. Life cycle inventory datasets for background processes were obtained from the ecoinvent v3.8 database. The authors did not collect new data.
Timing and spatial scale	All data are from publicly available sources documented in the main manuscript and the Supplementary Information file. Life cycle inventory datasets for background processes were obtained from the ecoinvent v3.8 database. The authors did not collect new data.
Data exclusions	No data were excluded from the analysis
Reproducibility	This study does not include experimental work
Randomization	The study does not include allocation of samples/organisms/participants into groups.

Blinding	Not relevant since all data used in this study are from existing datasets.

Did the study involve field work? ☐ Yes ☒ No

Reporting for specific materials, systems and methods

We require information from authors about some types of materials, experimental systems and methods used in many studies. Here, indicate whether each material, system or method listed is relevant to your study. If you are not sure if a list item applies to your research, read the appropriate section before selecting a response.

Materials & experimental systems

n/a	Involved in the study
☒	☐ Antibodies
☒	☐ Eukaryotic cell lines
☒	☐ Palaeontology and archaeology
☒	☐ Animals and other organisms
☒	☐ Clinical data
☒	☐ Dual use research of concern
☒	☐ Plants

Methods

n/a	Involved in the study
☒	☐ ChIP-seq
☒	☐ Flow cytometry
☒	☐ MRI-based neuroimaging

Plants

Seed stocks	Seed stocks were not collected in this study
Novel plant genotypes	Novel plant genotypes were not produced in this study
Authentication	N/A
